# Comparative Pathology and Pathogenesis of African Swine Fever Infection in Swine

**DOI:** 10.3389/fvets.2020.00282

**Published:** 2020-05-19

**Authors:** Francisco J. Salguero

**Affiliations:** Public Health England, Porton Down, Salisbury, United Kingdom

**Keywords:** African swine fever, pathology, pathogenesis, virus, swine

## Abstract

African Swine Fever (ASF) is a viral disease that affects animals of the *Suidae* family, and soft ticks from the genus *Ornithodoros* can also be infected by the ASF virus (ASFV). The disease was first described in Africa at the beginning of the twentieth century as an acute disease characterized by high mortality and fatal hemorrhages. ASF has caused outbreaks in numerous countries and it continues to be devastating nowadays for the porcine sector in those countries affected, and a massive threat for those free of the disease. ASF can follow clinical courses from peracute to chronic in domestic pigs (*Sus scrofa*) depending on a variety of factors, including the immune status of the animals and the virulence of the ASFV strain. The key features of the pathogenesis of the disease in domestic swine are a) a severe lymphoid depletion including lymphopenia and a state of immunodeficiency, and b) hemorrhages. However, African wild swine like bushpigs (*Potamochoerus larvatus*), red river hogs (*Potamochoerus porcus*), and warthogs (*Phacochoerus africanus*) can be infected by ASFV showing no clinical signs of disease and acting as natural reservoir hosts. In this article we review the key features of the gross and microscopic pathology together with a description of the pathogenesis of ASFV infection in domestic pigs following the different clinical courses. The pathogenesis of ASF in wild and domestic swine is also described, what can provide important information for the design of control strategies, such as vaccines.

## Introduction

African swine fever (ASF) is the most important infectious disease of swine and has proven to be devastating for the pork industry worldwide. ASF was first observed in the early 1900's in East Africa, when European domestic pig breeds were introduced in the Kenya Colony and animals developed a form of hemorrhagic disease with high morbidity and mortality (1). ASF was confined to African countries until 1957 when it reached Portugal via contaminated waste containing infected pork products that were used to feed local pigs. This outbreak was quickly controlled, but ASF re-entered Portugal in 1960 and spread rapidly to the Iberian peninsula (2) and produced sporadic outbreaks in several European countries, including Belgium, the Netherlands, Italy, Malta, and France (3–6). ASF spread to the Americas, with sporadic outbreaks in Brazil, the Dominican Republic, Haiti, and Cuba (7–11). ASF was eradicated from all these countries out of Africa, except the Italian island of Sardinia, where the disease has persisted since 1978 (2, 12–14). The disease continued to persist and spread within Africa (15) and entered the Republic of Georgia in 2007 through the port of Poti (16), most likely via contaminated food used to feed domestic pigs (17). ASF spread rapidly within the Caucasian region and neighboring countries and continues to spread to West, including European Union countries (18, 19) and to the East, with the disease causing abundant outbreaks and affecting dramatically the pork industry in China, Vietnam, Cambodia, Philippines, Laos, and East Timor (20–23).

ASF is caused by a large, complex, enveloped DNA virus (ASFV), from the family *Asfarviridae* (24). ASFV is composed of more than 50 structural proteins and can produce more than 150 proteins in the infected cells (17, 25–27), many of which are highly immunogenic. The main target cell for ASFV is the monocyte/macrophage in both domestic and wild swine (28–30), but infection in lymphocytes has not been reported (30). ASFV may also replicate in other cell types, including hepatocytes, renal tubular epithelial cells, neutrophils, and endothelial cells (31–33). The ASFV replication and the immune responses from the host induce different clinical courses and pathology in swine species. ASFV can also replicate in soft ticks from the genus *Ornithodoros*, including *O. moubata* in Africa and *O. erraticus* in the Iberian peninsula (34–37), which are involved in the epidemiological cycles of ASF (38, 39). Other soft tick species have also been reported to be susceptible to ASFV infection and may play a role in the epidemiology of ASF in other countries.

ASF has produced a high economic cost to the pork industry and it is the most important porcine disease nowadays, mostly due to the difficult prevention and control as no vaccine is available and other strategies must be used to control the disease from different territories. In this review article, we describe the different clinical and pathological features of ASF in domestic and wild suids together with the key pathogenic mechanisms that induce the disease in the host species.

## Clinical Presentation and Gross Pathology of ASFV Infection in Domestic Pigs

The clinical presentation and the gross pathological lesions of ASF in domestic pigs may vary depending on the virulence of the virus isolate, the route, and dose of infection and host characteristics (17). ASFV isolates can be classified as highly virulent, moderately virulent, and low virulent (40). The clinical courses observed in ASF in domestic pigs can be described as peracute (or hyperacute), acute, subacute, or chronic.

### Peracute ASF: Clinical Signs and Lesions

Highly virulent strains are typically responsible for this clinical course, characterized by a very rapid clinical course, with high fever (up to 42°C), anorexia, lethargy, and sometimes sudden death without signs of disease. This is often observed when the virus enters a naïve farm causing death of some animals before the explosion of clinical cases. Some animals can show respiratory distress due to the high fever, but no gross lesions are usually found at the *post mortem* examination.

### Acute ASF: Clinical Signs and Lesions

**Figure 1:**
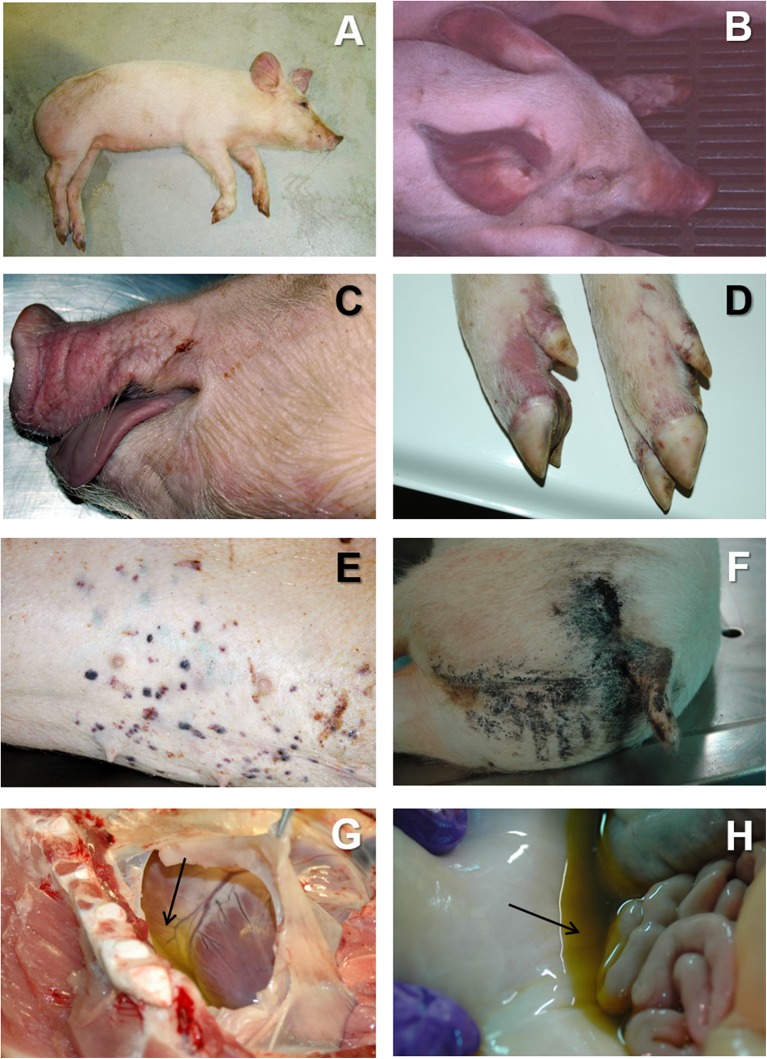
**(A)** Lethargic animal in acute ASF. The animal show cyanosis ion the ears abdomen and limbs. **(B)** Severe cyanosis in an animal suffering from acute ASF, associated to very high hyperthermia (41–42°C). **(C)** Cyanosis in the snout and lips in acute ASF. **(D)** Cyanosis in the limbs in acute ASF. **(E)** Multifocal petechiae and ecchymosis in the skin in acute ASF. **(F)** Blood-stained perianal area in a pig affected by subacute ASF. **(G)** Severe hydropericardium (arrow) in subacute ASF. **(H)** Moderate to severe ascites (arrow) in subacute ASF.

This clinical form is cause by highly or moderately virulent isolates, and it is the typical course observed in naïve farms very quickly after the first fatal cases are reported. The clinical course is characterized by high fever, with temperatures of 40–42°C, lethargy, anorexia, and inactivity (Figure 1A). The affected animals tend to bunch up together. Many affected animals show a centripetal cyanosis, easily found in the ears (Figure 1B), snout (Figure 1C), limbs (Figure 1D), abdomen, tail, and perianal area. Respiratory distress is usually observed, with severe pulmonary oedema in animals affected by highly pathogenic isolates (41, 42). Skin lesions are frequent, with presence of petechial hemorrhages or ecchymosis (Figure 1E). Other clinical signs may include nasal discharges, sometimes stained with blood (epistaxis), vomiting, and diarrhea, that can be also blood-stained (melaena) (17, 43–45), causing black-colored stains in the perianal area of the animal (Figure 1F). Abortions may occur in pregnant sows and the mortality rates may reach up to 100% in affected farms within 7 days of the onset of the disease.

**Figure 2:**
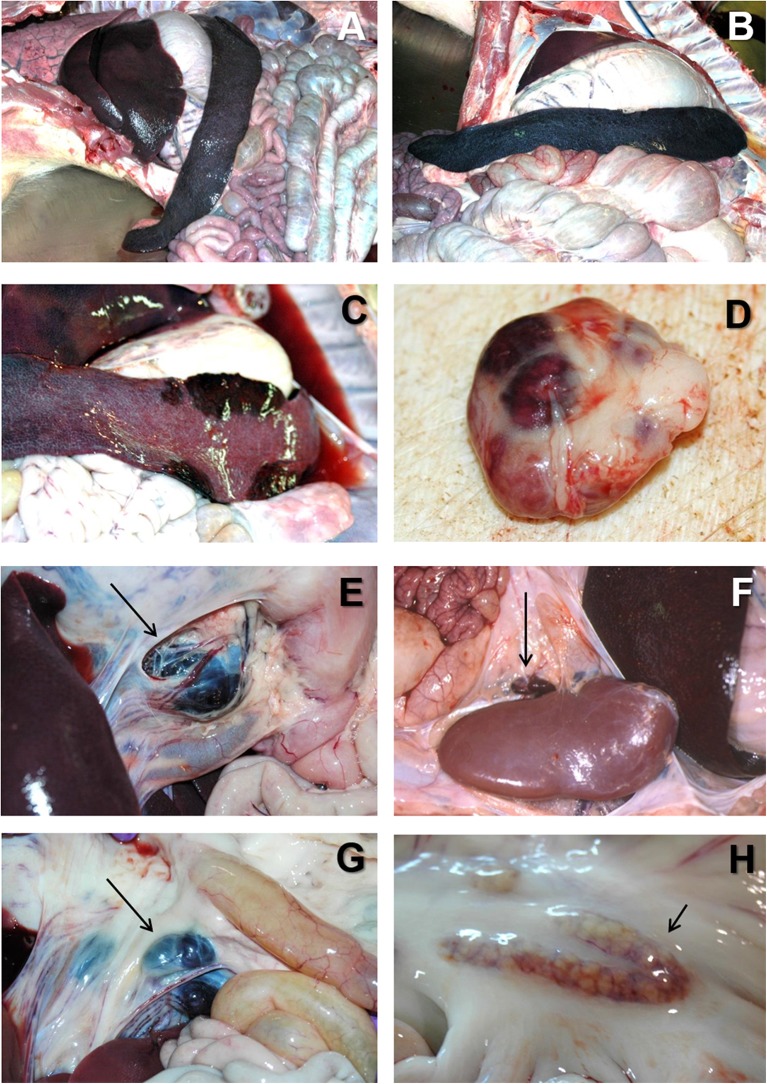
**(A)** Severe hemorrhagic splenomegaly observed at the opening of the abdominal cavity of an animal with acute ASF. The liver is severely congested. **(B)** Very large, dark colored spleen with rounded edges (hemorrhagic splenomegaly), and occupying a large volume of the abdominal cavity in acute ASF. **(C)** Multiple areas of partial hemorrhagic splenomegaly in the spleen from an animal with subacute ASF. **(D)** Multifocal hemorrhages in a lymph node with a marbled appearance in acute ASF. **(E)** Severe hemorrhagic lymphadenopathy in the gastrohepatic lymph node (arrow) in acute ASF. **(F)** Severe hemorrhagic lymphadenopathy in the renal lymph node (arrow) in acute ASF. **(G)** Severe hemorrhagic lymphadenopathy in the ileocaecal lymph node (arrow) in acute ASF. **(H)** Moderate hemorrhagic lymphadenopathy in the mesenteric lymph node (arrow) in acute ASF.

**Figure 3:**
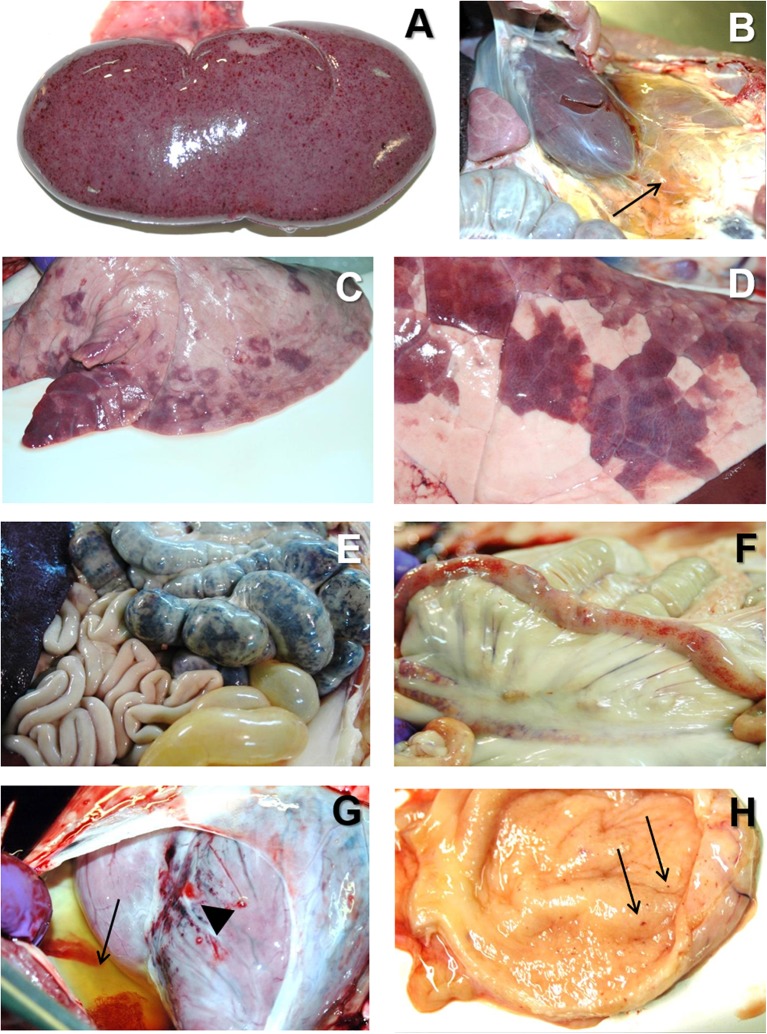
**(A)** Multiple petechial hemorrhages in the cortical surface of the kidney in acute ASF. **(B)** Severe perirenal oedema (arrow) in a pig with subacute ASF. **(C)** Multifocal areas of lung consolidation and pulmonary oedema in subacute ASF. **(D)** Multifocal pneumonia with dark color areas in the diaphragmatic lobe of the lung in subacute ASF. **(E)** Severe extensive hemorrhagic colitis in subacute ASF. **(F)** Multiple petechial hemorrhages in the serosa of the small intestine in acute ASF. **(G)** Multiple petechial ad ecchymotic hemorrhages in the epicardium (arrowhead) together with severe hydropericardium (arrow) in subacute ASF. **(H)** Multiple petechial hemorrhages in the mucosa of the urinary bladder in acute ASF.

At the *post mortem* examination, the most characteristic lesion of acute ASF is the hemorrhagic splenomegaly (28, 46, 47), with a very enlarged spleen, dark in color and friable at sectioning, occupying a large space within the abdominal cavity (Figures 2A,B). The second most important lesion described in acute ASF is a multifocal hemorrhagic lymphadenitis. Lymph nodes can have multifocal or extensive hemorrhages that can produce a marbled appearance (Figure 2D). The most affected lymph nodes are the gastrohepatic (Figure 2E), renal (Figure 2F), and other abdominal lymph nodes as ileocaecal (Figure 2G), and mesenteric (Figure 2H). Hemorrhages may also be observed with less frequency in other lymph nodes, such as submandibular, retropharyngeal, or inguinal. Petechial hemorrhages are often observed in the kidney surface (Figure 3A) and at sectioning. Other lesions can also be observed, mostly hemorrhages in the mucosa or the serosa of other organs, as the large (Figure 3E) and small intestine (Figure 3F), the epicardium in the heart (Figure 3G), or the urinary bladder (Figure 3H) (17, 43, 44, 48–51).

### Subacute ASF: Clinical Signs and Lesions

This clinical form is usually observed in animals infected by moderately virulent isolates, with similar clinical signs as those observed in acute ASF, although normally less marked (17). Affected pigs show moderate to high fever and the mortality rate ranges from 30 to 70% (17), with pigs dying at 7–20 after infection.

The vascular changes, mostly hemorrhages and oedema, in the subacute form of the disease can be more intense than the acute form (45, 52).

The death of affected animals may happen at two different stages: (a) during an initial thrombocytopenia and leukopenia (53–55), or (b) during a “recovery” phase, observed in young animals, causing erythrodiapedesis induced by vasodilation (53, 56).

At the *post mortem* examination, animals show hydropericardium (Figure 1G), ascites (Figure 1H), and multifocal oedema, very characteristic in the wall of the gall bladder or in the perirenal fat (Figure 3B) (17). Some animals may show hemorrhagic splenomegaly as described for the acute form of the disease, but many animals will show partial splenomegaly, with patches of spleen affected and other areas unaffected (Figure 2C). A multifocal hemorrhagic lymphadenitis can also be observed with multiple lymph nodes in all areas of the body showing the hemorrhages and the “marble” appearance (45). Petechial hemorrhages can also be observed in the kidney (50, 51). Multifocal pneumonia is also observed with patches of consolidation and dark color in the lung (Figures 3C,D). This lesion can also be attributed to secondary infections due to the state of immunosuppression induce by ASFV (45, 57, 58).

### Chronic ASF: Clinical Signs and Lesions

This clinical form is caused by the infection of low virulence isolates and has been observed, quite infrequently, in the Iberian Peninsula and the Dominican Republic (17, 54). It has been hypothesized that this low virulence isolates, and the associated chronic form, has evolved from ASFV isolates employed in early vaccine trials carried out in the Iberian Peninsula in the 1960's (17). The evolution of highly and moderately virulent isolates in other areas where the virus has been present for long periods of time has not produced this chronic form of the disease (17, 59).

This clinical form is characterized by multifocal necrosis in the skin and arthritis, growth retardation emaciation, respiratory distress and abortion (60, 61). No vascular changes are observed in the chronic form of ASF, and many observed lesions are associated with bacterial secondary infections, inducing fibrinous polyserositis, necrotic, or chronic pneumonia, necrosis of the skin, tongue, and tonsils (17, 43, 60).

## Pathogenesis of Lymphoid Depletion

ASF is characterized by severe leukopenia, mostly associated with lymphopenia, and a general state of immunodeficiency (58, 62). Initially, the virus enters the pigs following an oral-nasal route of after the bite of an infected soft tick. The virus replicates initially in the tonsils or regional lymph nodes (63, 64), spreading through the lymph and blood to secondary organs of replication within 2–3 days (65), and then spreading to the rest of the organs, where virus can replicate in a variety of cells (56, 66).

**Figure 4:**
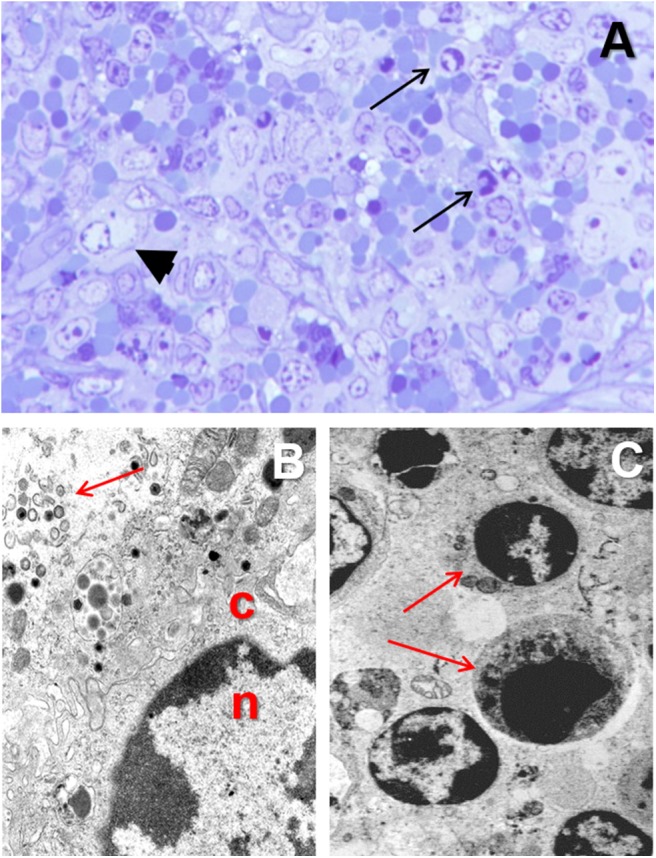
**(A)** Toluidine blue stained semithin (1 μm) section showing a macrophage with margination of the nuclear chromatin and a juxtanuclear clear intracytoplasmic inclusion body (arrowhead) in the spleen from a pig experimentally infected with acute ASF (3 dpi). **(B)** Transmission electron microscopy image of the nucleus (*n*) and cytoplasm (*c*) of a macrophage in the spleen from a pig infected with ASFV showing margination of the nuclear chromatin and a viral factory within the cytoplasm (arrow). **(C)** Apoptosis of lymphocytes (arrows) in the spleen of from a pig experimentally infected with acute ASF (5 dpi).

Monocytes and macrophages are the main target cell for ASFV (28, 42, 45). ASFV is a DNA virus, but the replication occurs within the cytoplasm and not in the nucleus (67–69). The infected monocyte-macrophage appears swollen, with margination of the nuclear chromatin (Figures 4A,B) and showing an intracytoplasmic juxtanuclear inclusion body, identifiable by its pale color when semithin (1-micron) sections are stained with toluidine blue dye (Figure 4A). These inclusion bodies show viral factories when studied under transmission electron microscopy (Figure 4B). The virus replication induce necrosis in the infected cells and virions are released by budding, and can be observed free in the blood, lymph, and the interstitial tissue (31, 70–72).

The destruction of monocytes-macrophages in ASF has been attributed to apoptosis (73) or necrosis (74) due to the action of ASFV (75). ASFV genome contain genes involved un programmed cell death both in an inhibitory or an inducing manner (64, 76–85). Some of these genes may promote the survival of the infected cells, and apoptosis has been described as the less likely cause of cell death in the infected monocyte-macrophage population (52, 58, 86).

ASF is characterized by a massive destruction of the lymphoid organs and tissues, including spleen, lymph nodes, thymus, and tonsils (58, 86, 87). There is a large proportion of B and T lymphocytes and macrophages undergoing cell death in acute ASFV infection (58, 78, 86, 88).

**Figure 5:**
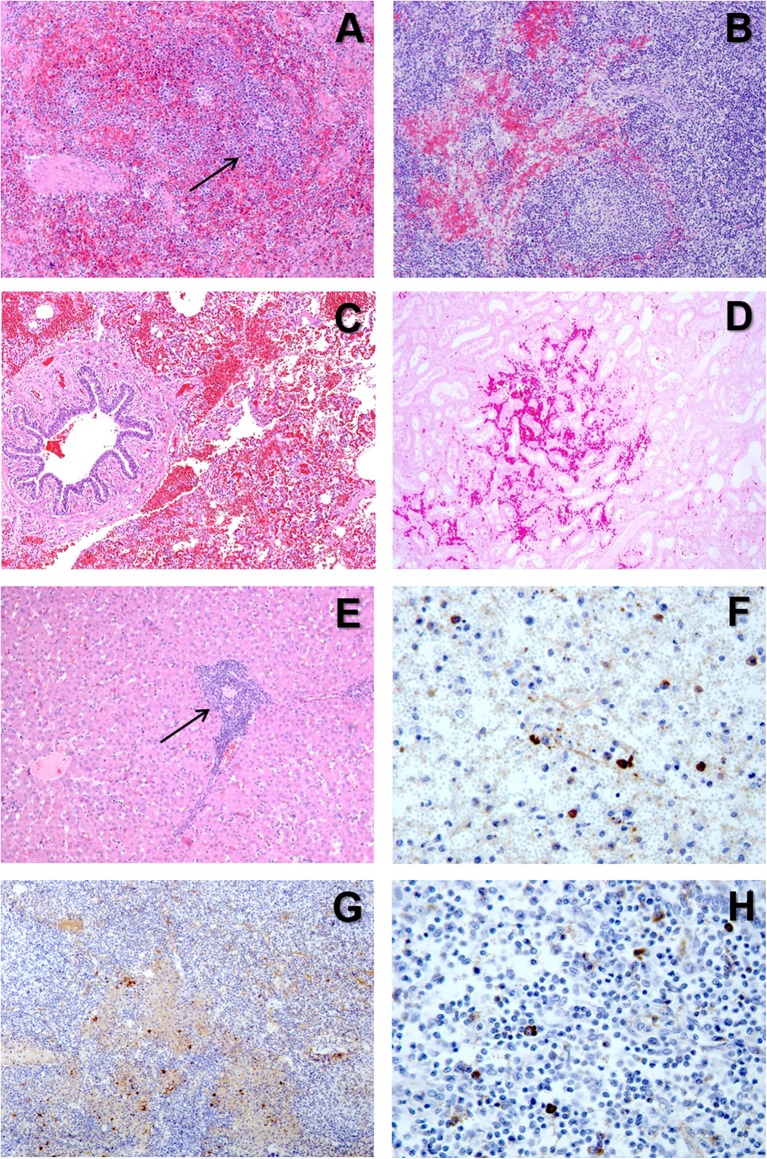
**(A)** H&E stain of the spleen from a pig with acute ASF showing abundant red blood cells within the red pulp and severe lymphoid depletion, with very small lymphoid follicles (arrow) in the white pulp. **(B)** H&E stain of the gastrohepatic lymph node from a pig with subacute ASF showing hemorrhages in the perifollicular lymphoid tissue and the medulla, together with a moderate lymphoid depletion. **(C)** H&E stain of the lung from a pig with subacute ASF showing severe hemorrhages in the septa and the alveolar spaces. **(D)** H&E stain of the kidney from a pig with acute ASF showing interstitial hemorrhages within the renal cortex. **(E)** H&E stain of the liver from a pig with acute ASF showing periportal inflammatory infiltrates (arrow) composed of lymphocytes, macrophages and plasma cells. **(F)** IHC detection of ASFV p72 in the spleen showing strong positive reaction in macrophages in the red pulp and cell debris within the necrotic areas. **(G)** IHC detection of ASFV p72 in the gastrohepatic lymph node showing strong positive reaction in macrophages within the perifollicular areas and the medulla. **(H)** IHC detection of ASFV p72 in the tonsil showing strong positive reaction in macrophages within the perifollicular areas.

The virus replication in the monocyte-macrophages (Figures 5F–H) induces an activation in this cell population and an increase in the secretion of proinflammatory cytokines have been observed at the early stages of the disease (28, 42, 58). The upregulation in the expression of proinflammatory cytokines, including IL-1, TNF-α, and IL-6, and described as a “cytokine storm” (89), is the responsible mechanism for the massive induction of apoptosis in lymphocytes (Figure 4C) neighboring the activated/infected monocyte-macrophages in tissues (58).

## Pathogenesis of Vascular Changes

ASF can be considered a hemorrhagic fever, with some pathogenic mechanisms similar to those described for hemorrhagic fevers affecting humans, as Ebola or Marburg filovirus infection (90, 91). Among the typical vascular changes observed in acute ASF, we can include petechial and ecchymotic hemorrhages in multiple organs, hemorrhagic, or hyperaemic splenomegaly, pulmonary oedema, and disseminated intravascular coagulopathy (D.I.C.). In subacute ASF, we can also observe these vascular changes together with a more marked oedema, ascites, and hydropericardium.

The most typical lesion in ASF is the hemorrhagic or hyperaemic splenomegaly (44, 46). The severity of this lesion will vary depending on the virulence of the isolate. The histopathological appearance of the spleen will include a hyperaemic red pulp, that can be completely filled with red blood cells (Figure 5A), platelet thrombi and cell debris, producing a disruption of the normal architecture of the organ (47, 58). The porcine splenic red pulp contains a mesh of fibers and smooth muscle cells surrounded by a population of macrophages fixed in the splenic cords (92). The necrosis of the macrophages in the red pulp is followed by a loss of intercellular junctions with the smooth muscle cells and the exposure of the basal lamina, inducing the activation of the coagulation cascade, platelet aggregation, and fibrin deposition, giving rise to the accumulation of red blood cells within the splenic cords (56, 93).

Hemorrhages are very common in the late phases of the disease, mostly in organs without a fixed vascular macrophage population, as the renal and gastrohepatic lymph nodes or the kidney (Figures 5B,D) (56). Even though ASFV can replicate in endothelial cells, this phenomenon has not been observed in all the organs showing hemorrhages (Figure 5C), and more importantly, this virus replication has only been reported in endothelial cells in the last phases of the disease, while hemorrhages may occur at earlier stages (33, 48). A different pathogenic mechanism has been observed and proposed as one of the main factors contributing to the hemorrhages in the early phases of the disease: the phagocytic activation of capillary endothelial cells, followed by endothelial cell hypertrophy that may lead to the total occlusion of the capillary lumen and a severe increase in the intravascular pressure (56). The subsequent loss of endothelial cells results in the exposure of the capillary basal membrane to which platelets can adhere, prompt the activation of the coagulation system and induce the D.I.C. (54–56).

An intense transient thrombocytopenia is frequently observed during subacute ASF, when hemorrhages are very frequent and severe (54, 55). This phenomenon may play an important role in the development of hemorrhages in the middle stages of the disease and is associated to structural changes of megakaryocytes in the bone marrow, with the presence of frequent denuded megakaryocytes (94), a feature also observed in relationship to hemorrhages in Classical swine fever (95).

The pathogenesis of the pulmonary oedema starts with the severe infection of pulmonary intravascular macrophages (PIMs), that is the main target cell for ASFV in the lung (31). Infected and non-infected PIMs tend to be enlarged and show signs of secretory activation. The production of proinflammatory cytokines such as IL-1α and TNF-α induce chemotactic activity and increase the endothelial permeability, leading to the leakage of fluid into the interalveolar septa and the alveolar spaces (42).

The marked anorexia in infected animals reduces dramatically the food/protein intake and accelerate the presence of hypo-oncotic oedema leading to internal fat consumption, ascites, hydrothorax, and hydropericardium, very typical in subacute ASF. Moreover, the liver of infected animals show a marked congestion, but also histopathological lesions, including multifocal periportal inflammatory infiltrates (Figure 5E), infection of Kupffer cells, which show severe secretory activation, and hepatocytes in the late stages of the disease (32, 49, 70, 96, 97). Hepatic malfunction may also contribute to the development of the multifocal oedema.

## ASF in the Eurasian Wild Boar

The Eurasian wild boar (*Sus scrofa*) is a native suid species of most of Europe and Asia and Northern Africa, but has also been introduced in other continents, including many islands. It is considered the natural ancestor of the domestic pig and both are classified as the same species. At present, the wild boar play a very significant role in the spread of ASF infection in Europe, and probably also in Asia, being also considered the main source of infection in the recent outbreaks in Central and Eastern Europe (98–102).

Due to the close taxonomic relationship between Eurasian wild boar and domestic pigs, many similarities in terms of immune responses to infections can be observed. However, even though they are the same species (*Sus scrofa*), they belong to different subspecies (101). Moreover, domestic pigs, and in some instances also wild boar, are managed with a close control on the health, reproduction and nutrition, whereas free-ranging wild boar are subjected to many natural variations on reproductive, sanitary, and nutritional conditions (101).

Before the outbreak of ASF in Georgia in 2007 and its further expansion, several studies were conducted to study the pathology and pathogenesis of ASFV infection wild boar, both in natural and experimental conditions [reviewed by Sanchez-Cordon et al. (101)]. No significant differences were found in the clinical presentation of ASF in wild boar compared with the domestic pig, with very similar acute, and subacute clinical courses, and associated lesions (17, 24, 103, 104). After 2007, a major emphasis has been put on the study of ASF in wild boar after the reports of infected individuals in relationship to the spread of the virus (105–109).

Several studies have been carried out in wild boars with low and high virulent isolates, in different settings and conditions. Highly pathogenic isolates from genotype II (110) induce hemorrhagic/hyperaemic splenomegaly, hemorrhagic lymphadenitis, pulmonary oedema, and multifocal petechial hemorrhages (64, 107, 111), sometimes described as even more severe than in the domestic pig (101). The mortality in is also very high (90–100%) in these infected animals. However, there are attenuated variants of the genotype II circulating in some parts of Europe (112–114). Infected wild boar with low virulent isolates and surviving the infection may transmit the virus to naïve contact animals for months, although current non-haemadsorbing genotype II isolates do not induce long-term carriers as a major outcome for recovery pigs isolates (111).

## ASF in African Warthogs and Bushpigs

In East Africa, ASFV is maintained in an ancient sylvatic cycle involving the common warthog (*Phacochoerus africanus*) and the arthropod vector (soft tick), *Ornithodoros moubata*, that inhabit their burrows (24, 85).

Since very early experimental studies, it was demonstrated that warthogs were very resistant to ASFV infection (1, 115), showing no clinical signs of the disease, except in young animals, which develop a transient viremia (116, 117). Viremia in adult warthogs is very rare with infectious virus mostly restricted to lymph nodes (85). The infectious ASFV may persist in warthog tissues for up to 25 weeks post infection, but is cleared by 56 weeks (118), what could explain the repeated re-infection of warthogs by ticks with the same virus strain (85).

Several genetics differences have been described between warthogs and domestic pigs (85). A difference between tolerance to infection and severe pathology may be due to a polymorphic RELA (p65; v-rel reticuloendotheliosis viral oncogene homolog A) variant found in warthogs (119).

ASFV has also been isolated from bushpigs (*Potamochoerus larvatus*) and red river hogs *(Potamochoerus porcus*), wild suid species found in sub-Saharan West and Central Africa (85, 116, 120, 121). ASFV infection does not induce clinical signs in these species, but moderate viremia can be observed (118, 120). ASFV can replicate in tissues without causing histological lesions, and mostly restricted to the B cell areas of the lymph nodes (85). Infected animals may transmit ASFV to feeding ticks but also to in-contact domestic pigs, although the role in the epidemiological maintenance of ASFV as a reservoir in unclear since these species do not inhabit burrows like warthogs and they are not in close contact with the *Ornithodoros spp*. ticks (85).

## Conclusions and Future Considerations

ASF is spreading very rapidly worldwide, and current control strategies rely on rapid detection, strict biosecurity, and implementation of quarantine and slaughter policies, in the absence of a commercial secure, and efficacious vaccine. These measures are not always implemented correctly or are insufficient, leading to culling large numbers of animals. The rapid detection is very important when ASF enters a new territory, and education, and communication are crucial tools to detect the first cases of the disease and follow up the official measures implemented to control the outbreaks. The clinical course and associated lesions of the disease may vary, and farmers and veterinarians must be always aware of the different presentations of ASF.

The pathogenesis of this disease is very complex, and more research is required to understand some of the pathogenic mechanisms, including how ASFV modulates the host immune responses and the role of the multiple proteins encoded by the virus. Several research groups are developing prototype vaccines mostly based on subunits or live attenuated isolates. More information is also needed to understand the correlates of protection to help with the development of these vaccines.

Finally, the presence of wild suids in the epidemiological cycles in Africa and Eurasia, makes the control of the disease very complicated, with the added problem of soft tick species as potential arthropod reservoirs in different countries. Moreover, the population of wild boar is increasing dramatically in Europe, but also in some parts of Africa and America, adding more problems to the control of ASF when outbreaks are reported. The rapid expansion of ASF in South Asia also raises the concern about the possibility of transmission into local wild suid species and the establishment of potential new epidemiological cycles in this and other areas of the world.

## Author Contributions

FS is the sole author of this manuscript, and conceived the idea of this review article after discussing ASF pathology with many colleagues in Asia during 2019, trying to produce a review focused on the pathology of ASF that could be useful to support veterinarians working in government and academic institutions, with abundant images and briefly discussing the main features of the disease in wild suids.

## Conflict of Interest

The author declares that the research was conducted in the absence of any commercial or financial relationships that could be construed as a potential conflict of interest.
